# X3DFast model for classifying dairy cow behaviors based on a two-pathway architecture

**DOI:** 10.1038/s41598-023-45211-2

**Published:** 2023-11-22

**Authors:** Qiang Bai, Ronghua Gao, Rong Wang, Qifeng Li, Qinyang Yu, Chunjiang Zhao, Shuqin Li

**Affiliations:** 1 Research Center of Information Technology, Beijing Academy of Agriculture and Forestry Sciences, Beijing, 100097 China; 2 College of Information Engineering, Northwest A&F University, Yangling, 712100 China; 3 National Innovation Center of Digital Technology in Animal Husbandry, Beijing, 100097 China

**Keywords:** Behavioural methods, Machine learning

## Abstract

Behavior is one of the important factors reflecting the health status of dairy cows, and when dairy cows encounter health problems, they exhibit different behavioral characteristics. Therefore, identifying dairy cow behavior not only helps in assessing their physiological health and disease treatment but also improves cow welfare, which is very important for the development of animal husbandry. The method of relying on human eyes to observe the behavior of dairy cows has problems such as high labor costs, high labor intensity, and high fatigue rates. Therefore, it is necessary to explore more effective technical means to identify cow behaviors more quickly and accurately and improve the intelligence level of dairy cow farming. Automatic recognition of dairy cow behavior has become a key technology for diagnosing dairy cow diseases, improving farm economic benefits and reducing animal elimination rates. Recently, deep learning for automated dairy cow behavior identification has become a research focus. However, in complex farming environments, dairy cow behaviors are characterized by multiscale features due to large scenes and long data collection distances. Traditional behavior recognition models cannot accurately recognize similar behavior features of dairy cows, such as those with similar visual characteristics, i.e., standing and walking. The behavior recognition method based on 3D convolution solves the problem of small visual feature differences in behavior recognition. However, due to the large number of model parameters, long inference time, and simple data background, it cannot meet the demand for real-time recognition of dairy cow behaviors in complex breeding environments. To address this, we developed an effective yet lightweight model for fast and accurate dairy cow behavior feature learning from video data. We focused on four common behaviors: standing, walking, lying, and mounting. We recorded videos of dairy cow behaviors at a dairy farm containing over one hundred cows using surveillance cameras. A robust model was built using a complex background dataset. We proposed a two-pathway X3DFast model based on spatiotemporal behavior features. The X3D and fast pathways were laterally connected to integrate spatial and temporal features. The X3D pathway extracted spatial features. The fast pathway with R(2 + 1)D convolution decomposed spatiotemporal features and transferred effective spatial features to the X3D pathway. An action model further enhanced X3D spatial modeling. Experiments showed that X3DFast achieved 98.49% top-1 accuracy, outperforming similar methods in identifying the four behaviors. The method we proposed can effectively identify similar dairy cow behaviors while improving inference speed, providing technical support for subsequent dairy cow behavior recognition and daily behavior statistics.

## Introduction

Accurate identification of dairy cow behaviors is a critical first step in analyzing and evaluating health status. This enables enhanced farm productivity and animal welfare. For example, breeders can determine estrus cycles by monitoring mounting behaviors, allowing timely artificial insemination to increase milk production. Additionally, analysis of standing and lying patterns indicates cow comfort and barn suitability. Automated behavior recognition greatly facilitates large-scale cattle monitoring, overcoming the need for extensive human observation. Overall, sophisticated computational models to automatically classify key cow behaviors provide actionable insights from behavioral data. These approaches hold promise for optimizing individual animal and herd outcomes through precise health evaluation, disease response, breeding management, and comfort assurance.

Deep learning is widely used in the animal behavior recognition field as a result of the rapid advancement of computer vision technology. Deep learning, as opposed to traditional methods of obtaining animal behavior information and recognizing behaviors using hardware devices such as pedometers^1,2^ and neck collars^3^, has the advantages of noncontact and high accuracy and can accurately recognize animal behaviors using only image and video data^4,5^. Liu et al.^6^ attached inertial measurement units (IMUs) behind the heads of 12 cows to collect cow behavior data. A fully convolutional network was utilized to extract behavioral features from the data and identify seven distinct behaviors-ruminating, lying, feeding, leg scratching, self-licking, neck scratching, and social licking-with over 80% accuracy. Similarly, Wu et al.^7^ established a deep residual bidirectional long short-term memory (LSTM) model using IMU data to recognize 7 cow behaviors: feeding, lying ruminating, social licking, and rub itching with high accuracy.

Convolutional networks are the foundation of deep learning and can automatically extract animal behavior features from images or videos^8^. This reduces the subjectivity of manual feature extraction and increases the effectiveness of feature extraction and animal behavior recognition. YOLOv5 was enhanced by Wang et al.^9^ to recognize dairy cow mounting behavior. Mounting behavior features were extracted in this study using GCBlock and the atrous spatial pyramid pool, and the model achieved an accuracy of 94.4%. Kang et al.^10^ used YOLOv4 and DenseNet to detect cow lameness. YOLOv4 was applied to locate the hoof. After removing background information and noise, the hoof information was entered into DenseNet to assess cow lameness; 92.39% and 98.50% were the model indicators. Chen et al.^11^ recognized pig-eating behavior with an accuracy of 98.4% using an Xception convolutional network. The studies mentioned above show that deep learning methods have been used for animal behavior recognition with positive results. The above research identifies a single animal behavior, and research on multiple animal behavior recognition is being conducted. Based on YOLOv5, Bai et al.^12^ identified six cow behaviors: standing, walking, mounting, lying, drinking, and eating. This study used an attention mechanism to improve the model's feature extraction and discrimination ability and achieved an average accuracy of 92.0%. The dairy cow walking behavior accuracy improved from 76.8% to 81.7%. Shang et al.^13^ used MobileNetv3 and the DeepSort tracking algorithm to recognize four cow behaviors: walking, standing, lying, and eating. DeepSort was used to analyze the trajectory changes in cows walking and standing to determine the two behaviors, and the accuracy reached 86.1%. Dairy cows standing and walking were more difficult to recognize using the cow behavior recognition model developed in the preceding study.

Prior work has utilized images for animal behavior recognition, but animal behaviors exhibit rich dynamic time-series features that cannot be fully characterized by single images alone, limiting the effective identification of behaviors such as walking and standing in dairy cows. Yin et al.^14^ extracted behavioral features from videos using EfficientNet and then fed the continuous features into long short-term memory (LSTM) to model the motor features of cow behaviors. The model was used to identify five behaviors: standing, lying, walking, drinking, and feeding. Its average recognition accuracy was 97.87%; for walking and standing, the accuracies were 99.0% and 96.9%, respectively. The usefulness of time-series data for identifying cow behaviors was confirmed by this study. The model was less robust because the cow behavior data used in this study, which consisted of simple scenes and obvious behavioral features, did not include dairy cow behaviors in obscured situations. A method combining LSTM and a feedforward neural network was suggested by Domun et al.^15^. Pigtail biting, diarrhea, and fouling are recognized as three different states by the model. The model's respective AUROC indicators are 1.782, 0.775, and 0.820. The study mentioned above used video data and trained a 2D model before adding a timing module to identify animal behaviors^16^. However, this approach has the drawbacks of a lengthy training period, reliance on the outcomes of each stage, and poor model performance.

The spatiotemporal information of animal behavior is directly modeled using 3D convolution in the proposed 3D convolutional model, enabling end-to-end model training to identify animal behaviors. Fuentes et al.^17^ identified 15 cow behaviors, including walking, standing, lying, and sleeping, using the I3D model to simulate cow behaviors. The method has an average accuracy rate of 85.6% for 15 behaviors due to the similarities between behaviors (such as lying and sleeping and walking and standing). The 3D model extension of RexNet by Ma et al.^18^ was able to recognize three cow behaviors: lying, standing, and walking. 3D-RexNet, which was directly extended to 3D, has an outstanding effect on cow behavior recognition with an accuracy rate of 95% due to the RexNet model's potent feature extraction capability. This study is comparable to a study by Yin et al.^14^, who used a less successful model and similar cow behavior data in a real farming environment. Previous studies on cow behavior recognition using videos, large model sizes and simple data resulted in slow inference and were unable to effectively identify cow behaviors on real farms. Therefore, we constructed a lightweight cow behavior recognition model and a dataset reflecting real cow behaviors for better generalization, facilitating deployment and use on different farms. Moreover, the lightweight nature of the proposed model allows rapid cow behavior recognition. By integrating analytical methods, it can provide daily behavior statistics on farms. Given sufficient historical data, the approach could analyze farm condition suitability and cow health status, supporting technological advancements in cattle welfare and farming practices.

As previously mentioned, the existing research on dairy cow behavior recognition suffers from a limited dataset, a large number of model parameters, and a slow inference process, making it unable to recognize dairy cow behaviors in actual farming settings. To address the aforementioned issues, the team first created a dataset of dairy cow behaviors that reflected a real-world farming setting. The 3D deep convolution method was then introduced and refined to create a lightweight model for the problem of a high number of 3D model parameters, and the X3DFast dairy cow behavior recognition model with a two-pathway architecture was proposed. The following are the paper's innovative points.

1. To enable the model to learn dairy cow behavioral motion patterns in real-world agricultural environments and improve model robustness, dairy cow behavior video datasets with varying instances of occlusion, video quality, camera angles, and clip durations were constructed.
2. A lightweight and efficient two-pathway X3DFast architecture was designed specifically for dairy cow behavior video classification.
3. The two pathways of X3DFast are improved by using a lightweight action attention model and R(2 + 1)D convolution to improve the model's accuracy in recognizing dairy cow behaviors.

## Related work

Behavior recognition has become one of the most important goals in computer vision research^19–21^. With the advancement of storage technologies that enable faster reading/writing speeds and larger capacities, researchers can record extensive behavioral data through video and provide solid data support for behavior recognition studies. In the field of deep learning for images, extracting image features using 2D convolutional neural networks has yielded satisfactory results. Because 2D convolution works on only a single image and cannot model temporal data to extract video features directly, researchers modified the model architecture to create 2D convolutional models with spatiotemporal modeling capabilities. Modeling the spatiotemporal features of a video using a 2D convolutional network and LSTM (Conv + LSTM) is the direct method^22^. The Conv + LSTM model architecture was successful in recognizing cow behavior^14^. The use of such designs, however, takes much time, according to other studies^23^. Two types of data, RGB streams and optical streams, were used in the implementation of 2D models for modeling video spatiotemporal features using dual-stream architectures^24,25^. The RGB stream provides spatial features, and the optical stream provides motion features. Wang et al.^26^ created a dual-stream network, called TSN, based on time slices using video data of varied lengths, employed a sparse sampling method for modeling long-term films, and provided a novel angle for further research. To rectify the observed behaviors, Zheng and Qin^27^ integrated the YOLOv5L model with a tracking method. This approach integrates static behavior detection with dynamic behavior correction, attaining a mean average precision of 88.4% for cow behavior recognition. Although they pruned YOLOv5L to reduce model parameters and improve detection speed, this two-stage approach with detection, tracking, and correction still requires extensive time. Since videos consist of continuous images, it is more logical to extend 2D convolutional operations to 3D convolutions for video analysis tasks. To learn the spatiotemporal features of objects, scenes and activities in videos, Tran et al.^28^ proposed a simple yet effective deep 3D ConvNet architecture. This 3D ConvNet can achieve strong performance on various video analysis tasks using just a linear classifier. Moreover, 3D-based models have been successfully applied for cow behavior recognition^18^.

The three traditional model architectures discussed above each have their strengths and weaknesses. For instance, 2D + SLTM models have a slow inference speed, and 3D models often contain a large number of parameters. Because of the limits of 2D convolution in temporal modeling, researchers have steadily preferred the study of 3D convolutional networks and suggested models that perform better^29–31^. To extract spatiotemporal features from videos for subsequent video-related tasks, Carreira et al.^23^ proposed a dual-stream inflated convolutional network, I3D, that inflates the convolution and pooling from the 2D convolutional model to 3D. In this study, the outcomes of the single-stream and two-stream I3D models were contrasted to demonstrate the superiority of the two-stream architecture of I3D. Feichtenhofer et al.^32^ proposed a 3D convolutional net with a two-pathway architecture that was motivated by the primate vision system. A SlowFast model with a two-pathway architecture was developed by integrating a slow pathway and a fast pathway. The fast pathway responds rapidly to temporal changes but is insensitive to spatial details and color information, while the slow pathway responds slowly to motion but captures fine-grained spatial details and color. Feichtenhofer et al.^33^ gradually expanded 2D convolutional networks from time, space, width, and depth to generate a series of effective and lightweight X3D convolutional models to address the issue of the large number of parameters in 3D convolutional networks and slow model inference and training. Motivated by previous work, this study integrates and enhances the strengths of the SlowFast and X3D models to develop a compact yet accurate two-pathway X3DFast architecture for dairy cow behavior recognition in practical agricultural environments.

## Dairy cow behavior dataset

Identifying dairy cow behavior was a major part of the study. With the consent of the farm owner, cameras were installed in parts of the cattle farm to obtain data on cow behavior; no actual contact with individual dairy cows occurred, and no experiments on live animals were carried out. The cameras used were Hikvision cameras without an infrared night vision function. We chose a fixed top-down view angle, tilted approximately 30° downwards, to record dairy cow behavior videos. The video resolution is 2560 × 1080 with 25 frames per second. The animal use protocol listed below has been reviewed and approved by the Ethics Committee, Northwest A&F University, China.

### Data sources

The data were collected from Beijing Dadi Qunsheng Dairy Cow Farm in October 2021, where dairy cow behaviors were regularly recorded using surveillance cameras. Following the screening, a dairy cow behavior video dataset of 4,366 videos of four types of dairy cow behaviors, lying, standing, walking, and mounting across, was created. The surveillance videos continuously recorded dairy cow movements on the farm over an extended period. As such, the number of dairy cows captured was variable over time rather than fixed. The number of dairy cows captured across the video dataset ranged from approximately 4 to 35. We decided to collect dairy cow behaviors from a variety of perspectives because dairy cow behaviors exhibit various behavioral features depending on the perspective. To improve the behavior dataset authenticity and restore the real dairy cow’s movement status, the video data of shading and multiple dairy cows were also kept in our constructed dataset.

### Dairy cow behavior video dataset

**Figure 1:**
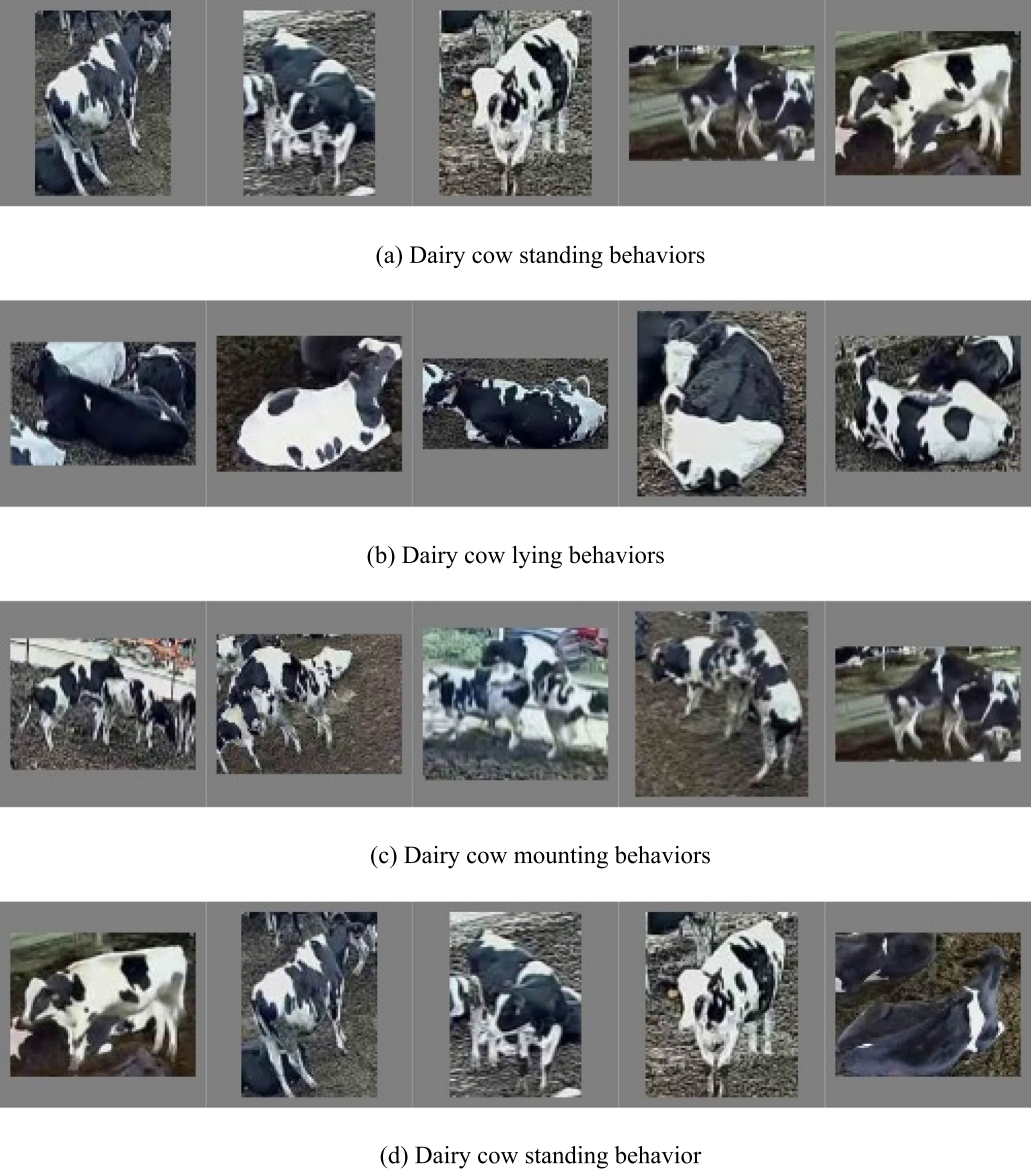
Example of dairy cow behavior data.

The video classification model extracts spatiotemporal features from the video to determine which category it belongs to. Individual dairy cows are manually cropped to create individual dairy cow behavior video datasets to identify cow behaviors, and four behavior samples are shown in Fig. 1.

Figure 1 depicts four examples of dairy cow behaviors: walking, standing, mounting, and lying. To preserve the multiscale features of dairy cow behaviors, the gray part expands the image to equal length and width. The figure also shows examples of challenging filming conditions, including varied illumination, blurring, and occlusion. This demonstrates how the dataset incorporates dairy cow behaviors across diverse background conditions, providing rich data for model training. The dairy cow subjects are more noticeable and less obscured in scenes with low density (Fig. 1a,b, etc.). The main body of the dairy cow is not always visible in scenes with high density, there are more instances of occlusion, other dairy cows are used as the background (Fig. 1c,a, etc.), and the blurring and dim lighting phenomenon is more noticeable at night. Due to occlusion, blurring, dim lighting, and visual similarity between foreground and background cows, recognizing individual dairy cow behaviors from videos is challenging for the model. Table 1 shows the criteria for judging dairy cow behaviors as well as details about the dairy cow behavior dataset.

**Table 1:** Dairy cow behavior dataset information.

Behaviors	Standing	Lying	Walking	Mounting
Behavioral expression	Dairy cow standing in video	Dairy cow sticking to the ground	Dairy cow walking in video	Two dairy cows overlapped
Number of videos	1799	1947	1130	774
Average video length	1-2s	1-2s	1-3s	2-4s

**Figure 2:**
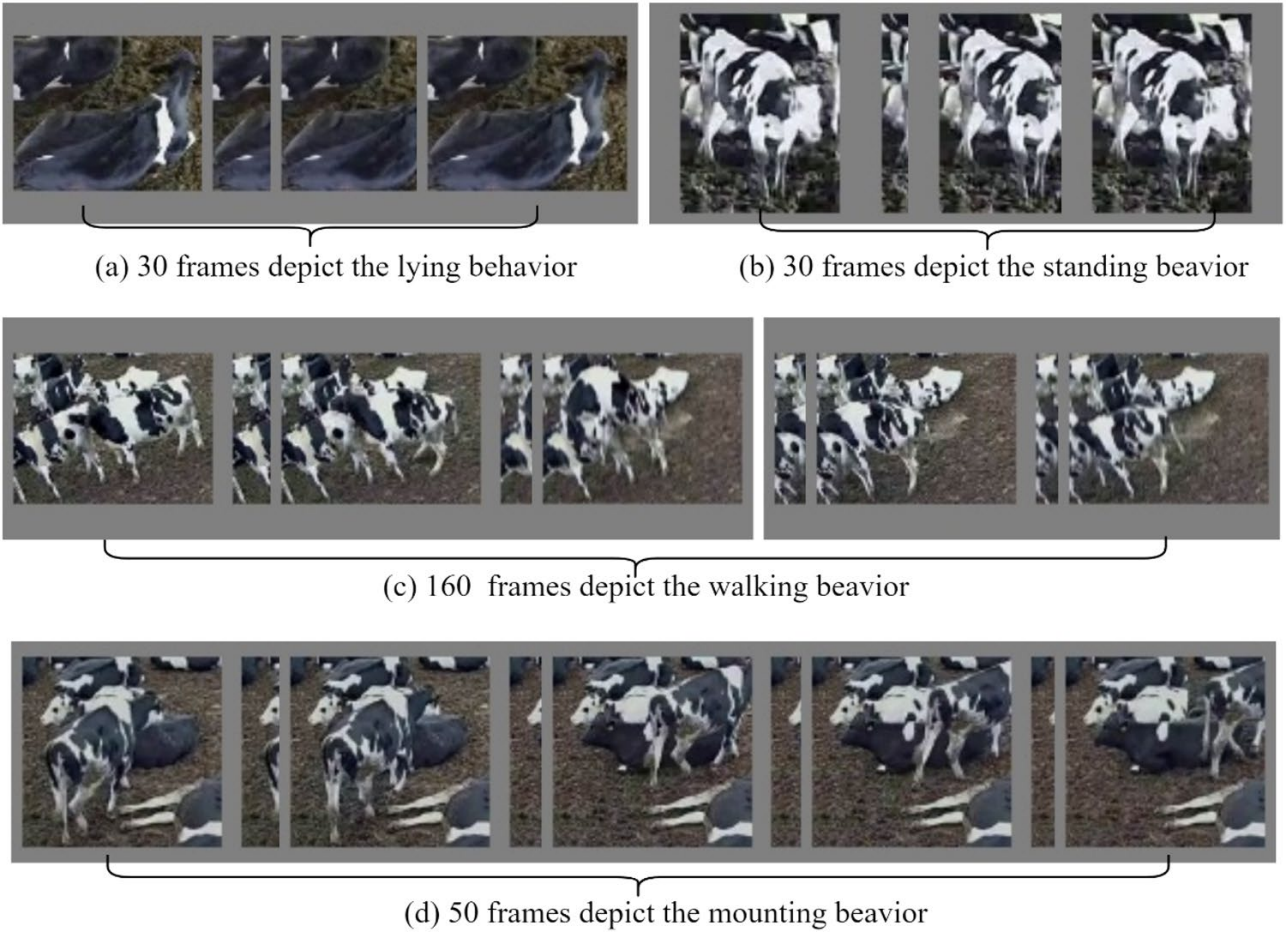
Comparison of frames needed for dairy cow behaviors.

As shown in Table 1, the dataset contains 1799 videos of standing behavior, 1947 videos of lying behavior, 1130 videos of walking behavior, and 774 videos of mounting behavior. The number of videos for each dairy cow’s behaviors varied. To ensure that the model can sufficiently learn features of different dairy cow behaviors, we adopted stratified sampling to split the training and test sets. For videos of each dairy cow’s behaviors, we randomly selected 80% of the videos to form the training set and the remaining 20% to form the validation set. Behaviors such as walking, standing and lying require only 1–3 s to express behavioral characteristics. However, dairy cows take a longer time to mount, so the mounting videos are between 2 and 4 s. The data in Table 1 demonstrate that mounting behaviors occurred with a low probability, while walking, standing, and lying behaviors dominated the daily dairy cow behaviors. As shown in Fig. 2, when building the dairy cow behavior dataset, the number of frames needed to convey information about dairy cow behaviors varied. The behaviors of standing and lying only needed a few frames to capture each cow action, whereas mounting behaviors had longer cycles that needed more frames to fully express the action in the videos. The different video lengths for each dairy cow behavior place greater demands on the model's capability to effectively model the temporal dimension. Additionally, the varying clarity and viewpoints of the videos place higher demands on the model's capacity to accurately spatially simulate dairy cow behaviors.

## Dairy cow behavior recognition model

**Figure 3:**
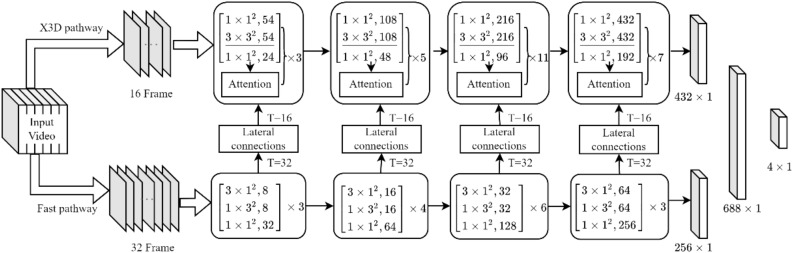
Schematic diagram of the structure of the improved X3DFast. *Note* The size of the convolution kernel is *T* × *S* × *S*, *C*, where T denotes the time-dimensional convolution size and *S* × *S* denotes the spatial-dimensional convolution size. Attention is the attention mechanism, DownSample(T) denotes downsampling in space, and FC is the fully connected layer. The underline indicates the use of depthwise separable convolution.

A new model, called the X3DFast model, was created by combining the SlowFast and X3D models. Based on features of different densities in dairy cow behavior data, similar foreground backgrounds, and different times needed for behavior expression, X3DFast is further improved to improve the model accuracy for dairy cow behavior recognition. Its structure is shown in Fig. 3.

The X3DFast architecture consists of four key components: the X3D pathway, the fast pathway, the lateral connection, and the predictor. The X3D pathway concentrates on static behavioral features in the spatial dimension, whereas the fast pathway concentrates on dynamic behavioral features in the temporal dimension. The 3D convolution samples down the feature map on the time dimension to match the feature dimensions of the X3D pathway and Fast pathway. The lateral connection method is improved to more accurately convey dairy cow motion features. The attention mechanism is incorporated within each residual block to fully extract the spatial features of dairy cow behaviors. Finally, the predictor, composed of fully connected layers, is fed the feature extraction results from the fast pathway and X3D pathway to predict the four dairy cow behaviors.

### X3D pathway

The X3D pathway is less dependent on the video frame rate and instead focuses on extracting spatial features of dairy cow behaviors. The X3D pathway structure is depicted in Fig. 1. There are four layers in total, with each layer corresponding to 3, 5, 11, and 7 residual convolution groups. The issue of feature loss during dairy cow behavior feature extraction can be solved by residual convolution groups. However, the 3D residual convolution group contains many parameters. To reduce the parameter quantity, the depthwise separable convolution, highlighted in Fig. 3, replaces the 3 × 3 × 3 3D convolution in the convolution group. To compensate for the reduced parameter quantity, an attention mechanism is incorporated after each convolutional group to enhance the feature extraction capabilities.

#### Action model

**Figure 4:**
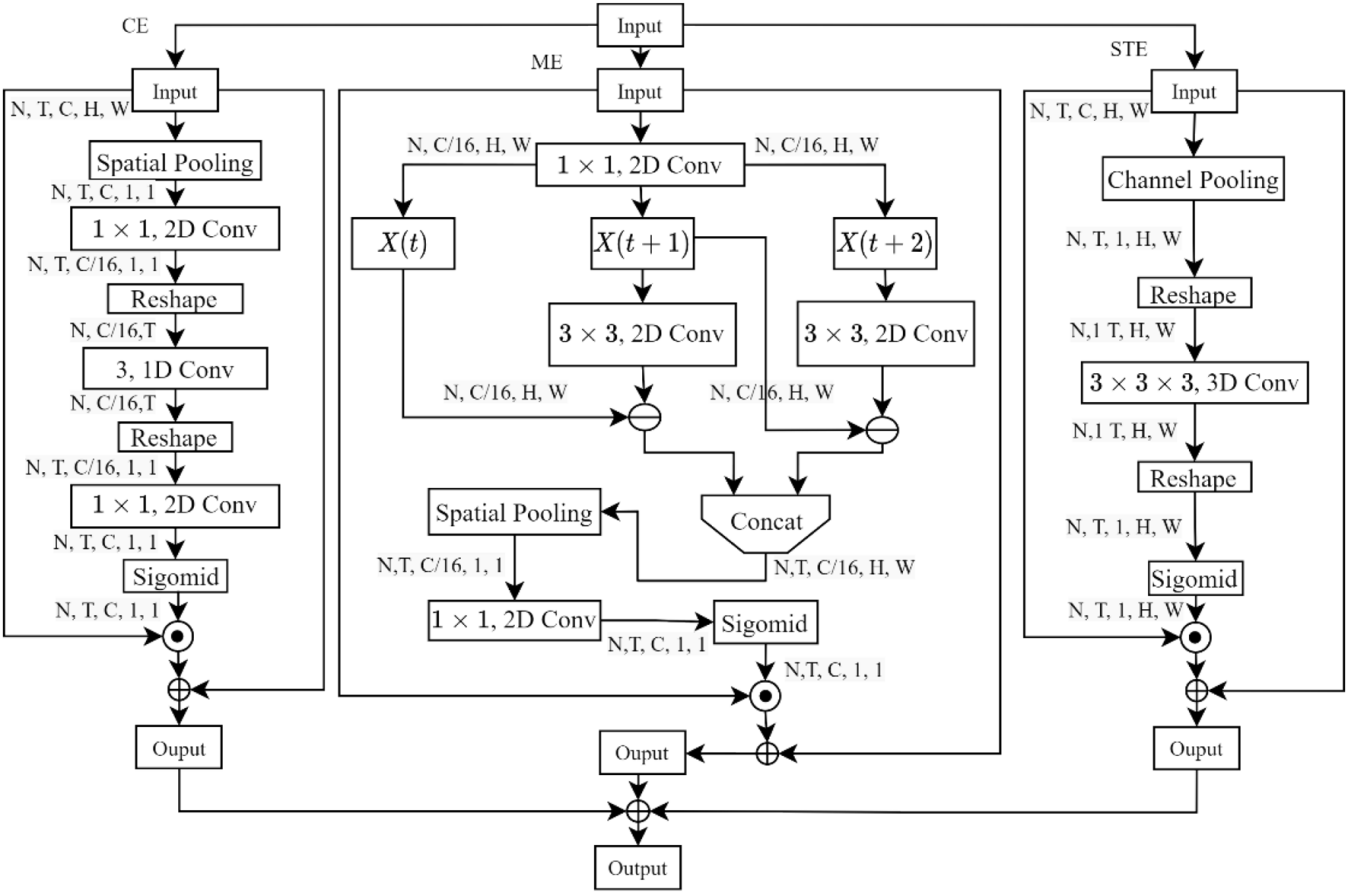
Diagram of the action model structure.

Typically, the 3D attention mechanism contains many parameters. The action model^34^ was used in this study to enhance the X3D pathway's capacity to model the spatial features of dairy cow behaviors without adding any additional parameters to the model. Its structure is shown in Fig. 4.

Spatial–temporal excitation (STE), channel excitation (CE), and motion excitation (ME) are the three branches of the action model. The STE describes the spatiotemporal features of dairy cow behaviors.

1 $$X^{\prime} = F_{S} \left( X \right)$$

2 $$F_{o} = F_{f} \left( {X^{\prime}} \right)$$

3 $$Mask = Sigmoid\left( {F_{o} } \right)$$

4 $$Y = X + X \cdot Mask$$

Analyzing the STE branch feature processing in conjunction with Fig. 4, the input feature is *X* ∈ R(*N* × *T* × *C* × *H* × *W*), and the output feature is *X'*(*N* × *1* × *T* × *H* × *W*). After a series of feature extraction operations with *F*_*f*_, *F*_*o*_ is obtained and fed into the sigmoid to generate a space–time mask (*Mask* ∈ *N* × *T* × *1* × *H* × *W*). *Mask* quantifies the importance of the three dimensions of frame, height, and width in dairy cow behavior data to the expression of dairy cow behavior features. To reconstruct the input feature, multiply *Mask* by* X* (Eq. 4).

The CE pathway shows the dependence between modeling passes in the temporal dimension. The operating equation of the adaptive calibration channel characteristic response is as follows.

5 $$X^{\prime} = \frac{1}{H \times W}\sum\limits_{i = 1}^{H} {\sum\limits_{j = 1}^{W} X } [:,:,:,,i,j]$$

6 $$F_{r} = F_{s} (X^{\prime})$$

7 $$F_{o} = F_{uns} (K(X^{\prime}))$$

The global receptive field *X'*(*N* × *T* × *C* × *1* × *1*) on the dairy cow behavior image is obtained after *X* is subjected to spatial pooling (Eq. 5), and this field is then processed using *F*_*s*_ and other operations to remove unnecessary information to produce *F*_*r*_, where *r* is the compression ratio. The 1D convolution K in Eq. (7) is used to represent the temporal features of dairy cow behaviors. A feature extension operation called *F*_*uns*_ maps X*'* to *F*_*o*_(*N* × *T* × *C* × *1* × *1*). The input features are reconstructed by passing *F*_*o*_ to the sigmoid function to create a *mask* and mapping it to *X* (Eqs. 3, 4).

As shown in the equations, the dynamic differences in various dairy cow behaviors are highlighted by modeling the motion information of adjacent frames (Eqs. 8 and 9).

8 $$F_{m} = K \times F_{r} [:,t + 1,:,:,:] - F_{r} [:,t,:,:,:]$$

9 $$F_{M} = [F_{m} (1), \ldots ,F_{m} (t - 1),0]$$

*F*_*r*_ represents the feature map of *X* following the 2D convolutional compression channel. After splicing adjacent frames *F*_*r*_, they are subtracted to obtain the information about the change in the dairy cow's attitude,* F*_*M*_ (*N* × *T* × *C/r* × *H* × *W*). K is a 3 × 3 convolution, and the value of the parameter 0 maintains the length *F*_*M*_ as *F*_*r*_. Equation (5) can be used to obtain the dynamic features of dairy cow behaviors (*N* × *T* × *C/16* × *1* × *1*) with a global receptive field, and Eqs. (3) and (4) can be used to map after a dimension change and to generate *Mask* using sigmoid.

The spatiotemporal features of dairy cow behaviors are jointly extracted by combining the action module, which is made up of the aforementioned three branches, from the perspectives of time and space, channel interdependence, and motion perception.

### Fast pathway

**Figure 5:**
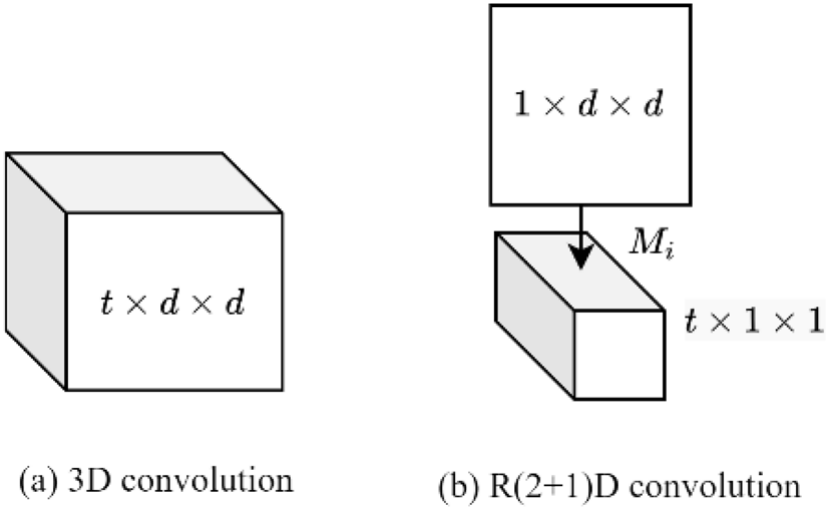
Schematic diagram of the R(2 + 1)D and 3D convolution structures.

Both the fast pathway and the X3D pathway were built using residual convolution, but they differ in the quantity of convolution and residual groups. The fast pathway is more inclined to extract temporal features of dairy cow behaviors due to having fewer convolutions and channels and a higher spatiotemporal resolution. To enhance the fast pathway's capacity for temporal modeling, R(2 + 1)D convolution^28^ was introduced in place of the original 3D convolution. Figure 5 depicts the R(2 + 1)D and 3D convolution structures.

Figure 5b shows the two input‒output operations of R(2 + 1)D. A 3D convolution of size *t* × *d* × *d* is shown in Fig. 5a, and the R(2 + 1)D convolution with the same parameters is shown in Fig. 5b. In the spatial dimension, the convolution size is *1* × *d* × *d*, and the convolution size in the temporal dimension is *t* × *1* × *1*. *M*_*i*_ is the number of channels in Fig. 5, and *M*_*i*_ (Eq. 11) is obtained to keep the same number of parameters for 3D convolution and R(2 + 1)D convolution, i.e., to satisfy Eq. (10). Figure 5a depicts a 3D convolution of size *t* × *d* × *d*, and Fig. 5b depicts an R(2 + 1)D convolution with the same parameters.

10 $$N_{i - 1} \times t \times d \times d \times N_{i} = N_{i - 1} \times 1 \times d \times d \times M_{i} + M_{i} \times t \times 1 \times 1 \times N_{i}$$

11 $$M_{i} = \left\lfloor {\frac{{td^{2} N_{i - 1} - N_{i} }}{{d^{2} N_{i - 1} + tN_{i} }}} \right\rfloor$$

The 3D convolution is divided independently into 2D spatial convolution and 1D temporal convolution by the R(2 + 1)D convolution. The decomposition operation doubles the nonlinear features compared to 3D convolution with an equivalent parameter quantity, enabling the model to fit more complex functions and learn the behavioral motion patterns of dairy cows. This facilitates model training and improves accuracy.

### Lateral connection

**Figure 6:**
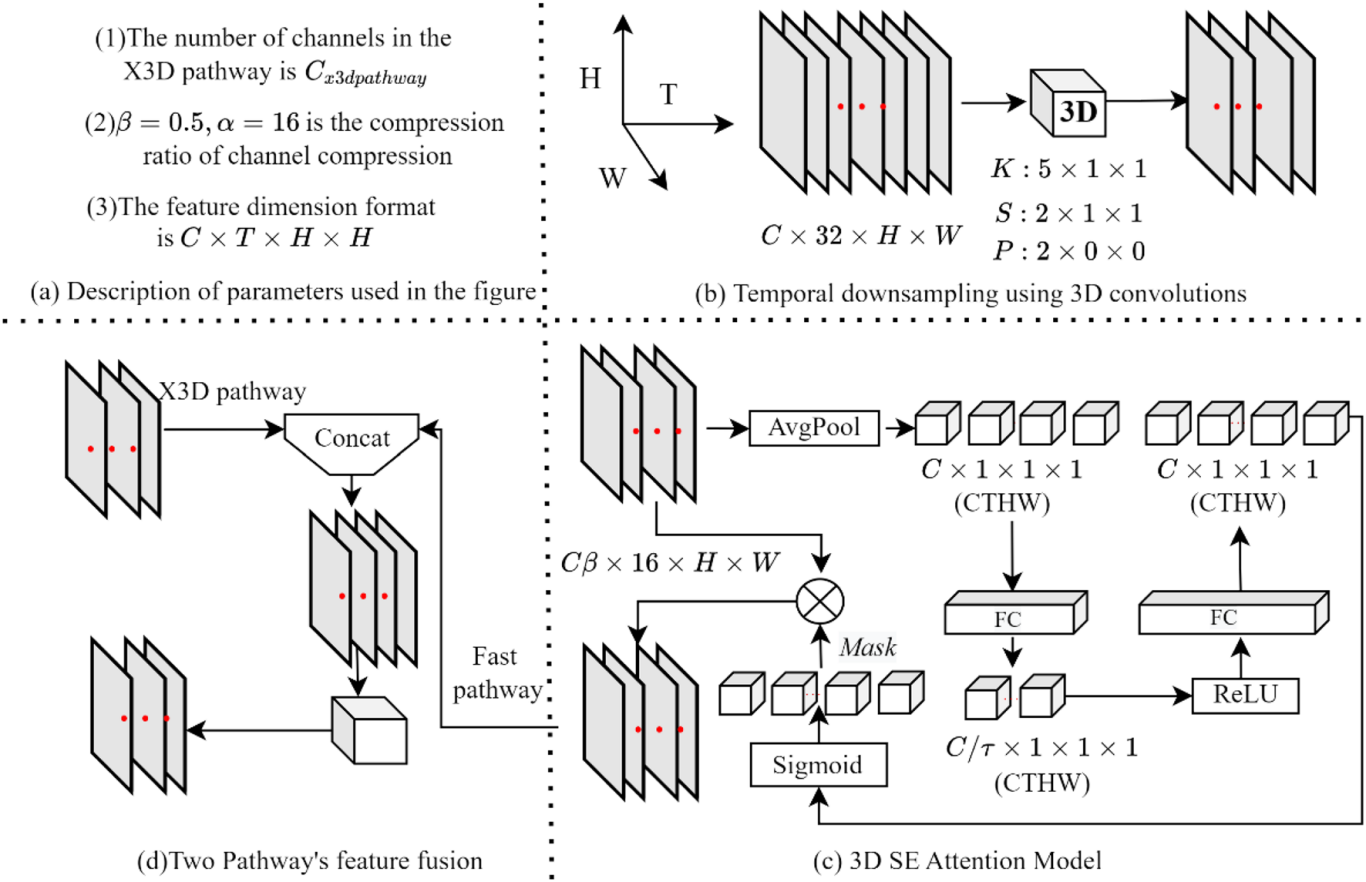
Schematic diagram of the lateral connection.

Through lateral connections, X3DFast transmits the motion features of dairy cow behaviors that were extracted by the fast pathway to the X3D pathway. Since the fast pathway and X3D pathway have different frame counts (*T*), stitching is performed after matching *T* using 3D convolution. Figure 6 depicts the lateral concatenation flow.

Figure 6 displays the four parts in Fig. 6a–d. The parameters used in Fig. 6 are described in Fig. 6a. A schematic representation of downsampling in the time dimension is shown in Fig. 6b. The 3D convolution parameters *K*, *S*, and *P* represent the kernel size, step, and padding, respectively. Figure 6c is the 3D SE attention model, and Fig. 6d is the fusion step.

A crucial step in integrating the X3D pathway and the fast pathway is lateral connection. The fast pathway's feature map is downsampled in the time dimension following 3D convolution to make the *T*_*Fast pathway*_ = *T*_*X3D pathway.*_ To improve the effect of lateral transmission, the 3D SE attention mechanism is used to reconstruct the timing features of dairy cow behaviors, and the dynamic features of representative dairy cow behaviors are given higher weights. Figure 6c depicts the 3D SE structure. As shown in Fig. 6d, the features reconstructed by 3D SE are spliced with the features of the X3D pathway to complete the lateral connection.

### Evaluate optimization metrics and loss functions

The cross-entropy function is commonly utilized to compute the loss for training machine learning models. The cross-entropy loss function is another training method the X3DFast model employs to close the gap between predicted and actual dairy cow behavior labels. Equation (12) is used to calculate loss.

12 $$L = \frac{1}{N}\sum\limits_{i} {L_{i} } = - \frac{1}{N}\sum\limits_{i} {\sum\limits_{c = 1}^{M} {y_{ic} } } \log \left( {p_{ic} } \right)$$

*M* is the number of dairy cow behavior categories (walking, standing, mounting, and lying) in the equation, and *y*_*ic*_ is a sign function. When the true category of sample *i* equals c, *y*_*ic*_ equals 1. *P*_*ic*_ is the probability that sample *i* belongs to category c. *L* is the loss, which is used to optimize the model's settings. Model performance is measured by X3DFast using the top-1 as in Eq. (13), where *y*_*ic*_ is the same as in Eq. (12).

13 $$Top - 1 = \frac{1}{N}\sum\limits_{i = 1}^{N} {y_{ic} }$$

## Dairy cow behavior recognition experiment

The experiments are performed on six 16 GB TeslaP100 GPUs with PyTorch1.1.7 and CUDA10.1. To demonstrate the effectiveness of X3DFast, dairy cow behavior video data are used to train the model, which is then compared to existing classic models (such as ResNet, I3D, and C3D). The training hyperparameters consisted of a learning rate of 0.01, 256 epochs, and stochastic gradient descent (SGD) as the optimization algorithm. The model was trained from scratch without using any pretrained weights. In the video experiments, the video resolution is set to 256 on the short side with reference to the parameter settings in the classical model and then randomly cropped to 224 × 224 video for propagation.

### Comparison with existing models

The performance of the existing video classification model for cow behaviors with single- and two-pathway architectures is shown in Table 2. According to the table, the X3DFast model has the best effect, and its model size is more advantageous than the others. X3DFast's top-1 value of 0.9849 in the two-pathway architecture is 0.1889 higher than SlowFast's top-1 value, despite the model size being only 10% of SlowFast's model size. When X3DFast is compared to single pathway architecture models, the top-1 of X3DFast is generally higher than the remaining single pathway architecture models; for example, the top-1 values of I3D and SlowOnly are 0.97 and 0.71 higher, respectively, while the model weights all differ by approximately 200 MB. Although the model size difference between X3DFast and X3D is smaller, the top-1 value of the X3DFast model is 0.62 percentage points higher than the top-1 value of X3D.

**Table 2:** Comparison with existing models. Significant values are in bold.

Model		Top-1	Model size	GFLOPs
Two pathways model	X3DFast	0.9849	**24.75**	15.93
	SlowFast	0.9796	256.7	36.44
Single pathway model	X3D	0.9787	**21.89**	12.94
	I3D	0.9752	208.1	43.56
	SlowOnly	0.9778	241.8	219.44
	C3D^29^	0.9805	321	–
	TSM_RGB_^35^	0.9840	179.79	5.38
	TSN_RGB_^26^	0.9273	179.79	5.38

Table 2 shows that the 3D model top-1 is generally higher than the 2D model, but the number of calculations is greater. The TSM_RBG_ backbone is ResNet50, and the TSM is trained using RBG images. When compared to the 2D model TSM_RBG_ and TSM_RBG_ results, the 2D model result is lower than the X3DFast's top-1, but the model inference speed is faster.

### Model training and results

**Figure 7:**
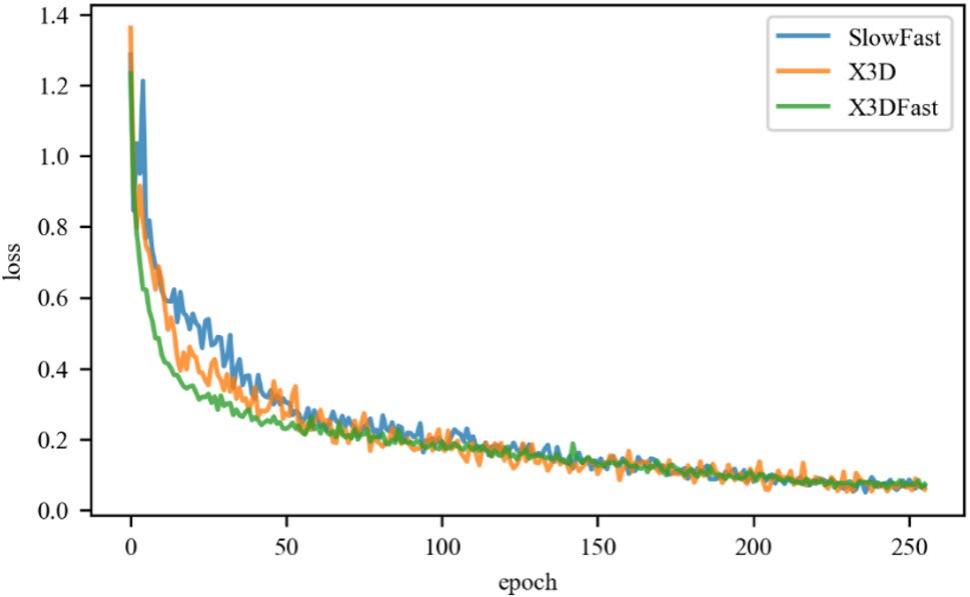
Model training loss comparison chart.

The loss function quantifies the effectiveness of model training and reflects how well the model fits the dairy cow behavior data. Figure 7 compares the training loss curves for the SlowFast, X3DFast, and X3D models.

**Figure 8:**
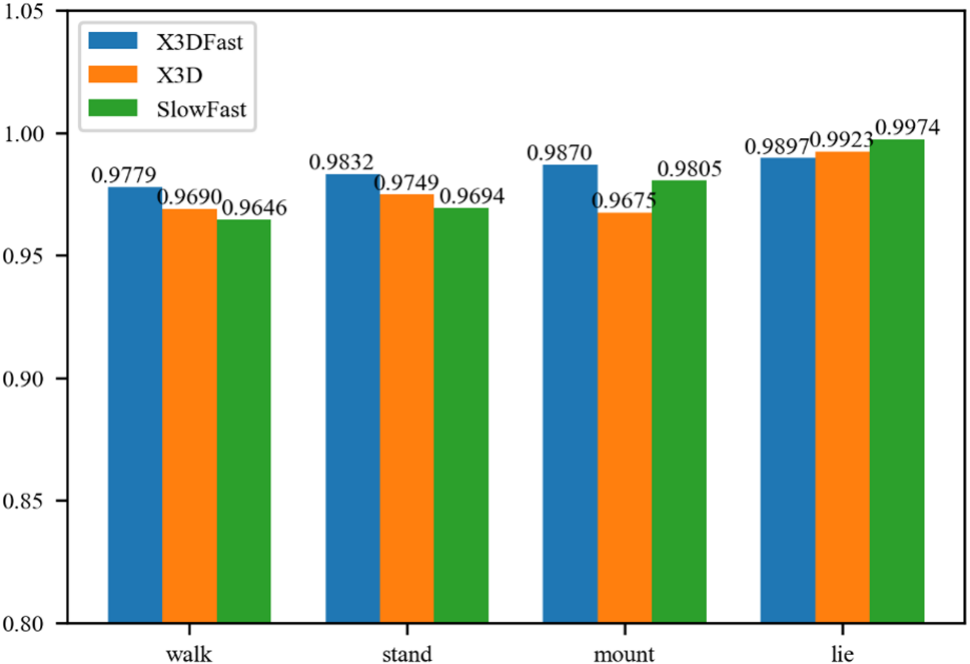
Individual dairy cow behavior recognition results.

As shown in Fig. 7, the training losses for SlowFast, X3DFast, and X3D exhibit a downward trend, decreasing continuously before plateauing and stabilizing over time. The loss of the X3DFast model decreases in the epoch range of 0–50 the quickest, while the loss of SlowFast and X3D decreases more slowly. This downward trend in training loss suggests that X3DFast is more effective at learning the behavioral traits of dairy cows compared to SlowFast and X3D. To evaluate this further, Fig. 8 compares the recognition results from the three models for different dairy cow behaviors.

The three models' identification results for dairy cow behaviors are shown in Fig. 8, and they are above 96%. The model produces high recognition rates for dairy cows that are mounting and lying but low recognition rates for those that are standing and walking. Dairy cows’ standing and lying behaviors are relatively stationary along the time dimension. Therefore, the model relies more on spatial features to distinguish these behaviors. This results in better recognition performance, as the image background is less likely to change drastically for stationary behaviors. It is challenging to identify the dairy cow behavior model because the spatiotemporal dimension features of dairy cow mounting and walking behaviors are constantly changing, but X3DFast also produces promising results. From the perspective of a single behavior, the model's ability to recognize dairy cow mounting behaviors is greater than its ability to recognize dairy cow standing behaviors because of the time features it has learned to distinguish between the two behaviors.

### Ablation experiments

Ablation experiments are conducted to investigate the impact of various improvement strategies on the performance of the X3DFast dairy cow behavior recognition model. Table 3 presents the experimental outcomes.

**Table 3:** Ablation experiments.

Models	Lateral connection	R(2 + 1)D	Action	Top-1
X3D + Fast				0.9787
X3D + Fast + latern	√			0.9796
X3D + Fast + R(2 + 1)D	√	√		0.9814
X3D + Fast + Action	√		√	0.9823
X3DFast	√	√	√	0.9849

All of the study's improvement techniques, as shown in Table 3, have the potential to enhance the dairy cow behavior recognition model's performance. The top-1 increased by 0.09% following the X3DFast model's lateral connection method optimization. In the fast pathway, the original 3D convolution is replaced by the R(2 + 1)D convolution, which also separates the spatiotemporal features of dairy cow behaviors, allowing various pathways to gain more useful features of dairy cow behaviors. Top-1 is enhanced by this strategy by 0.27%. Top-1 rose by 0.36% after the action model attention mechanism was added to the X3D pathway. When the abovementioned improved methods are combined to build the X3DFast, the model's top-1 improves by 0.62%.

### CAM of dairy cow behaviors

The 3D convolution in X3DFast enables the model to focus on regions that effectively capture the behavioral features of dairy cows. This subsection utilizes class activation maps to visualize the important areas that were focused on by X3DFast.

**Figure 9:**
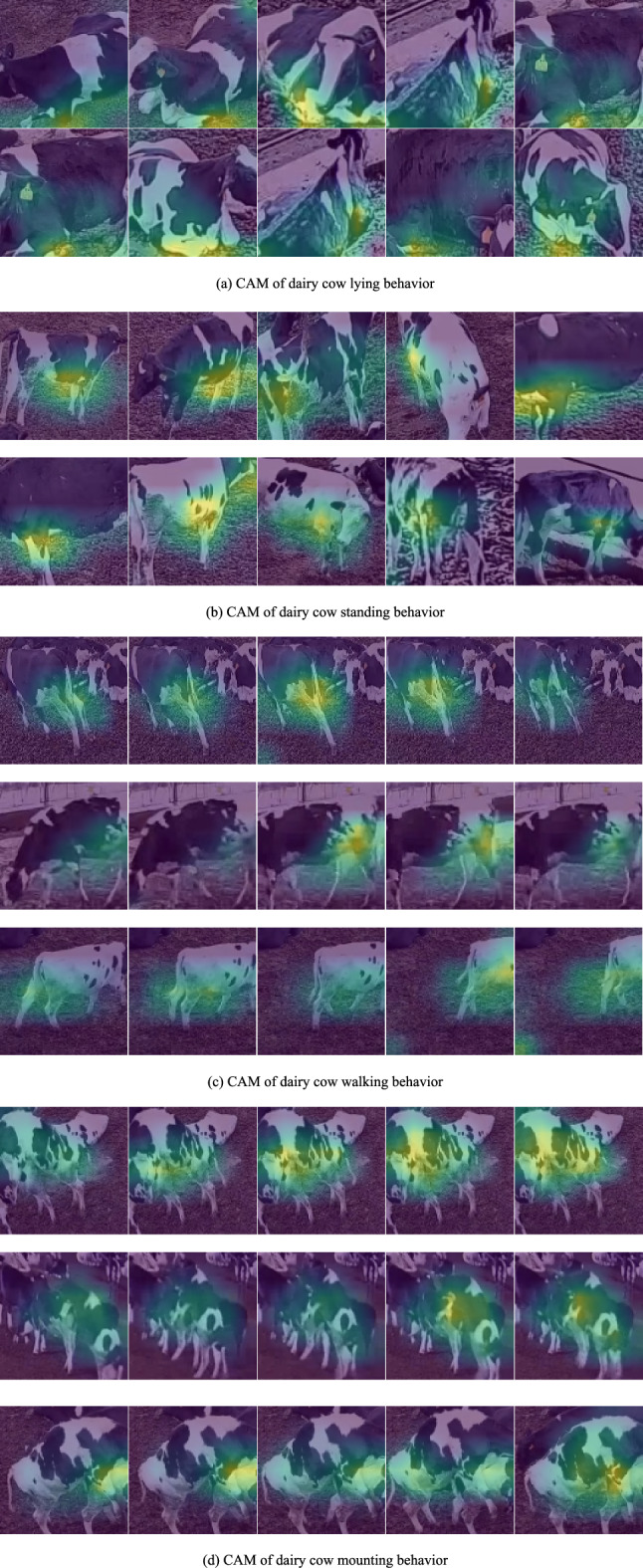
CAM of X3DFast dairy cow behaviors.

The CAM plots of the four dairy cow behaviors are shown in Fig. 9, and the brightly colored section of the plot represents the region of interest to X3DFast, i.e., the model determines the features of this region to be representative of the dairy cow behaviors. Figure 9a,b show CAM photos of dairy cows lying and standing in various positions while being obscured by various objects. The dairy cow leg areas are the bright areas in Fig. 9a,b, and the traits of this area are representative traits of lying and standing dairy cow behaviors. The continuous frame photos in Fig. 9c demonstrate that X3DFast maintains focus on the dairy cow's leg region during the dairy cow's walking motion. The model interprets the local spatiotemporal features as representing dairy cow behaviors. The model focuses on the area where the two dairy cows intersect during the mounting process so that the dairy cow mounting behaviors may be predicted (Fig. 9d).

In conclusion, X3DFast demonstrates an ability to focus on the most informative regions for recognizing the four dairy cow behaviors. Additionally, even with multiple cows in the background, X3DFast appears able to concentrate on the key aspects needed to distinguish behaviors.

### Comparison with other studies

This section compares the results of our study to other related works on dairy cow behavior recognition, as presented in Table 4. We summarized related articles on cow behavior recognition from 2022 to 2023. Table 4 shows that cow behavior recognition research can be divided into two main approaches: computer vision-based and hardware sensor-based. We intuitively illustrate the relevant contents and results of these studies in the table.

**Table 4:** Compared to related studies.

Studies	Method	Research content	Result (%)	Model size (MB)	GFLOPs
Liu et al.^6^	Accelerometer	Multibehavior recognition	Accuracy: 83.75	–	–
Brouwers et al.^1^	Accelerometer	Multibehavior recognition	Accuracy: 70.50	–	–
Zheng et al.^36^	Computer vision	Cow lameness detection	Accuracy: 94.37	–	–
Lodkaew et al.^8^	CowXNet	Estrus behavior detection	Accuracy: 83.0	–	–
Wu et al.^37^	YOLCAT + LSTM	Multibehavior recognition	Accuracy: 93.56	–	–
Wang et al.^2^	Accelerometer	Estrus behavior detection	Accuracy: 90.91	–	–
Hosseininoorbin et al.^37^	Accelerometer	Multibehavior recognition	Accuracy: 89.30	–	–
Wang et al.^9^	YOLOV5	Estrus behavior detection	mAP: 94.30	164	–
Xiao et al.^38^	Mask R-CNN + SVM	Cow recognition	Accuracy: 98.67	–	–
Qiao et al.^16^	C3D-ConvLSTM	Multibehavior recognition	Accuracy: 90.32	–	–
Ma et al.^18^	Rexnet3D	Multibehavior recognition	Accuracy: 95.00	14.3	15.80
Yin et al.^14^	EfficientNet + LSTM	Multibehavior recognition	Accuracy: 97.87	21.28	–
ours	Computer vision	Multibehavior recognition	Top1: 98.49	24.75	15.93

These related works recognize different cow behaviors using various methods. Since the studies differ in recognition approaches and research aims, their experimental results cannot be directly compared. However, for similar methods, the results using the same evaluation metrics can be simply compared. Studies without relevant comparable metrics are marked '-' in the table. The Method column shows the models or techniques used, with 'Accelerometer' indicating accelerometer data collection followed by machine learning for behavior recognition. The research content covers multibehavior recognition, estrus recognition, and individual identification based on differing research goals. Consequently, the studies utilized different evaluation metrics, such as accuracy, mAP, and top-1. The model size and GFLOPs are also shown, reflecting the computational requirements.

The data show that Liu et al.^6^ and Brouwers et al.^1^ used different accelerometers to obtain time-series behavior data and recognized behaviors using SVM. However, due to different behavior classes and numbers, the accuracy varies greatly. Qiao et al.^16^, Ma et al.^18^, and Yin et al.^14^ used 3D computer vision for behavior recognition, allowing a more direct comparison. Their results are 90.32%, 95.00% and 97.87%, respectively. Although the data types used are different, it can still be seen from the data in the table that the image-based method has a significantly higher accuracy in identifying cow behavior than the accelerator-based method. However, image-based methods are often susceptible to the influence of image background and lighting, which will lower the accuracy of cow behavior recognition. Cow behavior data obtained with accelerators contain less noise information, so accelerator-based methods are more generalizable.

The methods by Qiao et al.^16^, Ma et al.^18^, and Yin et al.^14^ are similar to ours in terms of methodology, but there are still differences. In terms of data, we retained the occlusion characteristics in the dairy cow behavior video data, which allows the model to have better generalization. In terms of methodology, X3DFast extracts spatial and temporal features of dairy cow behavior from the constructed X3D pathway and fast pathway, considering both the static and dynamic characteristics of dairy cow behavior. Therefore, it can achieve better recognition results on more complex data. We used different metrics. Top-k is for multiclass models, and usually, k = 1 or 5. When k = 1, the accuracy of the proposed model equals the accuracy obtained in the above studies, and hence, the methods are comparable. The data show that our X3DFast scores surpass the above results.

## Conclusion

This study suggested an environment-applicable multiangle X3DFast dairy cow behavior recognition model. The technique obtains an accuracy of more than 97% for all individual dairy cow behaviors and a top-1 index of 98.49% for recognizing four dairy cow behaviors: walking, standing, mounting, and lying. In conclusion, the X3DFast dairy cow behavior recognition model developed in this study has higher advantages in model size and inference speed, and it can recognize dairy cow behaviors in various lighting and perspective conditions, which can serve as a guide for future multitarget dairy cow behavior identification. The goal of our research is to develop an efficient and fast dairy cow behavior recognition method. As shown by the experimental results, we achieved the current objective. In addition, we plan to utilize multiobject tracking algorithms combined with the X3DFast model constructed in this paper to achieve tracking and recognition of cow behaviors in surveillance videos. That is, we plan to establish a one-to-one correspondence between individual cows and their behaviors across all dairy cow behavior videos, enabling more refined dairy cow behavior recognition. This work is currently underway, building upon the X3DFast model. After completing this work, we can monitor changes in dairy cow behaviors on farms more precisely, improving economic benefits and breeding welfare.

### Practical implications

In terms of data, this study deployed cameras in breeding environments to obtain reliable cow behavior datasets. The dataset contains instances of occlusion, varying lighting conditions, etc., to reflect real farm environments, and relatively complete cow behaviors were recorded. Therefore, the model can still stably and reliably recognize behaviors even when the video content varies greatly.

In terms of algorithms, the cow behavior recognition model constructed in this paper is lightweight and accurate. The model has low computational complexity and can process cow behavior videos to recognize behaviors without relying on high-performance GPUs.

From a practical application perspective, the model is easy to deploy and has high accuracy, which are significant advantages. After the model recognizes cow behaviors, it can determine cow behaviors over a period. By analyzing these data, we can judge whether the cow breeding environment is satisfactory. For example, if the walking behavior data for a day have a much larger fluctuation compared to the past, it may indicate changes in the breeding environment. Moreover, if mounting behavior data increases noticeably, it can indicate to breeders to increase the monitoring of cows in that area to quickly identify cows in heat for mating.

In summary, fast and accurate cow behavior recognition is very important for both the economic benefits of cow breeding and cow welfare.

## Data Availability

The data that support the findings in this study are available from the corresponding author, but restrictions apply to the availability of these data, which were used under license for the current study, and so they are not publicly available. The datasets used and analyzed during the current study are available from the corresponding author upon reasonable request (Ronghua Gao).

## References

[1] Brouwers SP, Simmler M, Savary P, Scriba MF (2023). Towards a novel method for detecting atypical lying down and standing up behaviours in dairy cows using accelerometers and machine learning. Smart Agric. Technol..

[2] Wang J, Zhang Y, Bell M, Liu G (2022). Potential of an activity index combining acceleration and location for automated estrus detection in dairy cows. Inf. Process. Agric..

[3] Krieger S (2018). Prediction of calving in dairy cows using a tail-mounted tri-axial accelerometer: A pilot study. Biosyst. Eng..

[4] Chen C, Zhu W, Norton T (2021). Behaviour recognition of pigs and cattle: Journey from computer vision to deep learning. Comput. Electron. Agric..

[5] Wang R (2022). Detection method of cow estrus behavior in natural scenes based on improved YOLOv5. Agriculture.

[6] Liu M (2023). Classification of cow behavior patterns using inertial measurement units and a fully convolutional network model. J. Dairy Sci..

[7] Wu Y (2022). Recognising cattle behaviour with deep residual bidirectional LSTM model using a wearable movement monitoring collar. Agriculture.

[8] Lodkaew T, Pasupa K, Loo CK (2023). CowXNet: An automated cow estrus detection system. Expert Syst. Appl..

[9] Wang R (2022). Oestrus detection in dairy cows by using atrous spatial pyramid and attention mechanism. Biosyst. Eng..

[10] Kang X, Li S, Li Q, Liu G (2022). Dimension-reduced spatiotemporal network for lameness detection in dairy cows. Comput. Electron. Agric..

[11] Chen C (2020). Recognition of feeding behaviour of pigs and determination of feeding time of each pig by a video-based deep learning method. Comput. Electron. Agric..

[12] Bai Q (2022). Multi-scale behavior recognition method for dairy cows based on improved YOLOV5s network. Trans. Chin. Soc. Agric. Eng. Trans. CSAE.

[13] Shang C, Wu F, Wang M, Gao Q (2022). Cattle behavior recognition based on feature fusion under a dual attention mechanism. J. Vis. Commun. Image Represent..

[14] Yin X, Wu D, Shang Y, Jiang B, Song H (2020). Using an EfficientNet-LSTM for the recognition of single Cow’s motion behaviours in a complicated environment. Comput. Electron. Agric..

[15] Domun Y, Pedersen LJ, White D, Adeyemi O, Norton T (2019). Learning patterns from time-series data to discriminate predictions of tail-biting, fouling and diarrhoea in pigs. Comput. Electron. Agric..

[16] Qiao Y, Guo Y, Yu K, He D (2022). C3D-ConvLSTM based cow behaviour classification using video data for precision livestock farming. Comput. Electron. Agric..

[17] Fuentes A, Yoon S, Park J, Park DS (2020). Deep learning-based hierarchical cattle behavior recognition with spatio-temporal information. Comput. Electron. Agric..

[18] Ma S, Zhang Q, Li T, Song H (2022). Basic motion behavior recognition of single dairy cow based on improved Rexnet 3D network. Comput. Electron. Agric..

[19] Laptev & Lindeberg. Space-time interest points. In *Proceedings Ninth IEEE International Conference on Computer Vision*, Vol. 1 432–439. 10.1109/ICCV.2003.1238378 (2003).

[20] Le QV, Zou WY, Yeung SY, Ng AY (2011). Learning hierarchical invariant spatio-temporal features for action recognition with independent subspace analysis. CVPR.

[21] Over, P. D. *et al.* TRECVID 2013: An overview of the goals, tasks, data, evaluation mechanisms, and metrics. *NIST* (2014).

[22] Ng, J. Y.-H. *et al.**Beyond Short Snippets: Deep Networks for Video Classification*. 10.48550/arXiv.1503.08909 (2015).

[23] Carreira, J. & Zisserman, A. *Quo Vadis, Action Recognition? A New Model and the Kinetics Dataset*. 10.48550/arXiv.1705.07750 (2018).

[24] Feichtenhofer, C., Pinz, A. & Zisserman, A. *Convolutional Two-Stream Network Fusion for Video Action Recognition*. 10.48550/arXiv.1604.06573 (2016).

[25] Simonyan, K. & Zisserman, A. *Two-Stream Convolutional Networks for Action Recognition in Videos*. 10.48550/arXiv.1406.2199 (2014).

[26] Wang, L. *et al.**Temporal Segment Networks: Towards Good Practices for Deep Action Recognition*. 10.48550/arXiv.1608.00859 (2016).

[27] Zheng Z, Qin L (2023). PrunedYOLO-Tracker: An efficient multi-cows basic behavior recognition and tracking technique. Comput. Electron. Agric..

[28] Tran, D. *et al.* A Closer Look at Spatiotemporal Convolutions for Action Recognition. Preprint at 10.48550/arXiv.1711.11248 (2018).

[29] Tran, D., Bourdev, L., Fergus, R., Torresani, L. & Paluri, M. Learning Spatiotemporal Features with 3D Convolutional Networks. In *2015 IEEE International Conference on Computer Vision (ICCV)* 4489–4497. 10.1109/ICCV.2015.510 (2015).

[30] Tran, D., Ray, J., Shou, Z., Chang, S.-F. & Paluri, M. *ConvNet Architecture Search for Spatiotemporal Feature Learning*. 10.48550/arXiv.1708.05038 (2017).

[31] Xie, S., Sun, C., Huang, J., Tu, Z. & Murphy, K. *Rethinking Spatiotemporal Feature Learning: Speed-Accuracy Trade-offs in Video Classification*. 10.48550/arXiv.1712.04851 (2018).

[32] Feichtenhofer, C., Fan, H., Malik, J. & He, K. *SlowFast Networks for Video Recognition*. 10.48550/arXiv.1812.03982 (2019).

[33] Feichtenhofer, C. *X3D: Expanding Architectures for Efficient Video Recognition*. 10.48550/arXiv.2004.04730 (2020).

[34] Wang, Z., She, Q. & Smolic, A. *ACTION-Net: Multipath Excitation for Action Recognition*. 10.48550/arXiv.2103.07372 (2021).

[35] Lin, J., Gan, C. & Han, S. TSM: Temporal shift module for efficient video understanding. In *2019 IEEE/CVF International Conference on Computer Vision (ICCV)* 7082–7092. 10.1109/ICCV.2019.00718 (2019).

[36] Zheng Z, Zhang X, Qin L, Yue S, Zeng P (2023). Cows’ legs tracking and lameness detection in dairy cattle using video analysis and Siamese neural networks. Comput. Electron. Agric..

[37] Hosseininoorbin S (2021). Deep learning-based cattle behaviour classification using joint time-frequency data representation. Comput. Electron. Agric..

[38] Xiao J, Liu G, Wang K, Si Y (2022). Cow identification in free-stall barns based on an improved Mask R-CNN and an SVM. Comput. Electron. Agric..

